# Long‐Range Spin Transport in Chiral Gold

**DOI:** 10.1002/adma.202506523

**Published:** 2025-06-11

**Authors:** Tapan Kumar Das, Offek Marelly, Shira Yochelis, Yossi Paltiel, Ron Naaman, Jonas Fransson

**Affiliations:** ^1^ Department of Chemical and Biological Physics Weizmann Institute Rehovot 76100 Israel; ^2^ Applied Physics Institute Center for Nanoscience and Nanotechnology Hebrew University of Jerusalem Jerusalem 91904 Israel; ^3^ Department of Physics and Astronomy Uppsala University Uppsala 752 21 Sweden

**Keywords:** chirality, Hall effect, interconnect, spin

## Abstract

Any attempt to use spintronics‐based logic elements will need to have spin interconnects to transfer information between its elements. Typically, the mean free path of an electron's spin in metals, at room temperature, is of the order of tens to hundreds of nanometers. Here chiral gold films are used to demonstrate that spin information can be transferred to distances of several microns at room temperature. The conduction of spins is accompanied by a Hall effect that exists without applying an external magnetic field. It is verified that the spin diffusion length is consistent with the frequency‐dependent Hall effect which indicates a spin‐effective lifetime in the order of nanoseconds. A theoretical model is presented that involves the anisotropic electronic polarizability of the system, its spin–orbit coupling, and spin exchange interactions.

## Graphical abstract

It is demonstrated that in chiral gold film, spin information can be transferred to distances of several microns at room temperature. The conduction of spins is accompanied by the Hall effect that exists without applying an external magnetic field. The spin diffusion length is consistent with the frequency‐dependent Hall effect which indicates spin effective lifetime on the order of nanoseconds.

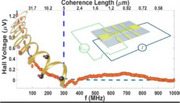

## 1 Introduction

Finding materials that enable spin interconnection in spintronics is essential for producing electron spin‐based integrated electronics.^[^ ^1^, ^2^, ^3^, ^4^ ^]^ The desire is of course to have interconnects at room temperature. Magnetic materials were proposed for this application but integrating them into the spintronics’ logic is complex and they may affect the nearby spintronic devices. Graphene was also suggested as a possible material for spin interconnects, however, implementing it in a conventional production process raises major challenges.^[^ ^5^ ^]^ The chiral‐induced spin selectivity (CISS) effect introduces the possibility of using chiral material for spin transport.^[^ ^6^ ^]^ When spin‐polarized electrons are injected into non‐magnetic metals at room temperature, the electron's spin population is expected to be equalized between the two spin states within tens of nanometers. In chiral organic and inorganic crystals, it was found that spin randomization occurs over distances that may exceed many microns.^[^ ^7^, ^8^, ^9^ ^]^ However, many of those chiral systems are incompatible with the microelectronics production process and have high resistance.

Here we present chiral gold film as a possible spin‐interconnect. Producing chiral gold nanoparticles and films was demonstrated by seeding the growth of the metal with chiral molecules^[^ ^10^ ^]^ similar to the production of chiral oxide in biomineralization.^[^ ^11^ ^]^ We show that the spin diffusion length in chiral gold at room temperature is long‐range and accompanied by a topological anomalous Hall (TAH) effect that can also be utilized in spintronics. Frequency‐dependent studies reveal a long effective spin lifetime which can be explained based on the chiral‐induced spin selectivity (CISS) effect.^[^ ^12^ ^]^

In the conventional Hall effect, the electric potential is measured perpendicular to the direction of the electric current. This is a result of applying a magnetic field perpendicular to the plane in which current is flowing. In general, obtaining Hall signals requires breaking time reversal symmetry (TRS). Another type of Hall effect is the anomalous Hall effect.^[^ ^13^ ^]^ In this case, the current flows through a material that has unpaired electrons and is ferromagnetic, or the spins in the material are polarized, and as a result, TRS breaks due to asymmetric spin scattering. It occurs in solids with broken time‐reversal symmetry, namely in a ferromagnetic phase, for example, as a consequence of spin–orbit coupling.^[^ ^14^ ^]^ A third type of Hall effect is the topological Hall effect which was found, for example, in static skyrmion lattices due to a scalar spin chirality.^[^ ^15^ ^]^ Recently it was shown both theoretically and experimentally that the topological Hall effect can also emerge dynamically from thermal spin fluctuations.^[^ ^16^ ^]^

Hall signals, without an external magnetic field, were also observed in achiral conductors or semiconductors that do not have unpaired electrons but were coated with chiral molecules. Their signal was associated with the anomalous Hall effect.^[^ ^17^, ^18^ ^]^

Here we report that the long‐range spin diffusion length through chiral gold is accompanied by the observation of the *topological anomalous Hall* (TAH) effect which results from spin‐polarized electron transport through a non‐magmatic chiral material and spin‐selective scattering of the electrons from polarized atoms. It is observed without applying an external magnetic field. The effect is a dynamic effect in a nonmagnetic system. The experimental observations are followed by a theoretical model that involves the CISS effect.^[^ ^19^ ^]^

## 2 Experimental and Results

**Figure 1:**
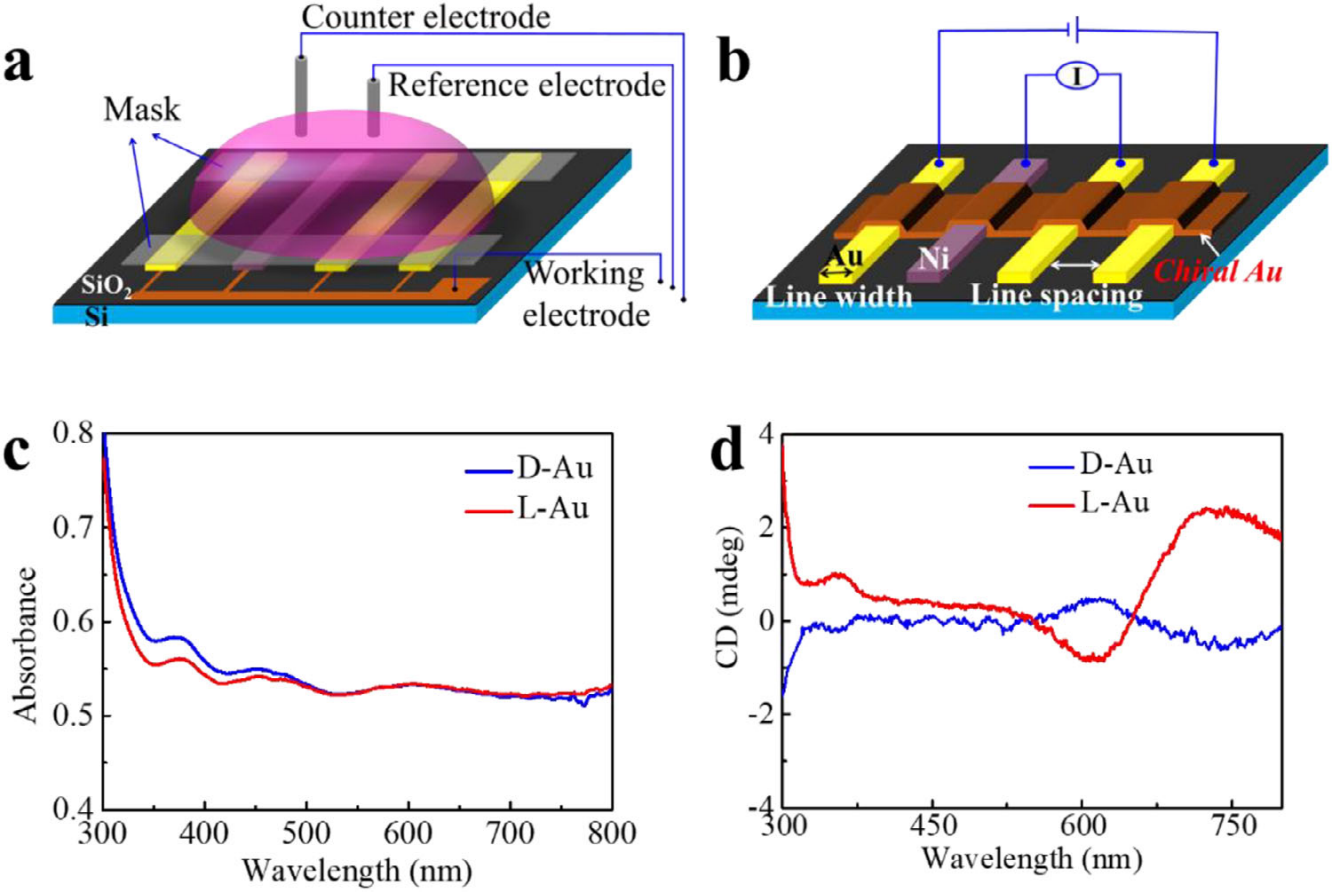
The device and its characterization. a) Schematic of the experimental setup used for electrochemical deposition of Chiral Au. All four electrodes are connected in parallel and serve as working electrodes. A Pt wire was used as the counter electrode (CE), Ag/AgCl/KCl_sat_, was the reference electrode (RE). b) The experimental setup for the magnetoresistance measurements. c) The absorption spectra of the chiral gold layer. d) The CD spectra of the chiral layers. The CD spectrum differs from that of pure tartaric acid and arises from the chiroptical response of the gold film induced by the chiral molecules during the growth of the gold film in the presence of L‐/D‐tartaric acid. The peak at ≈370 nm is associated with the interband electronic transitions of gold, which typically occur in the UV–vis region. The peaks at ≈600 and ≈750 nm are attributed to the plasmonic resonance modes of the chiral nanostructures or chiral domains formed within the film.

We prepared chiral gold film in a process that is based on electrodeposition of gold from an electrolyte containing gold salt (Na_3_Au(S_2_O_3_)_2_) and either L or D tartaric acid.^[^ ^20^, ^21^ ^]^ The chirality of the film was determined by circular dichroism spectroscopy for both enantiomers (**Figure** **1**d). The gold film thickness was ≈150 nm. The absorption spectra of the chiral gold are presented in Figure 1c. It is important to appreciate that the CD spectra appear in the gold absorption region, namely, that the imbedded chiral molecules induce chirality in the structure of the amorphous film which is made from gold nanoparticles. Two experimental setups were applied. In the first, the spin dependent transport was measured by a four‐contact device (Figure 1b; Figure S1, Supporting Information) when the voltage was applied between the outermost pair of electrodes and the inner pair of electrodes used to measure the current. One of the inner electrodes is made from nickel and for the spin measurements, it is magnetized by an external magnetic field of 1 T parallel or antiparallel to the current. The distance between the two internal electrodes was changed between 2 and 6 µm.

**Figure 2:**
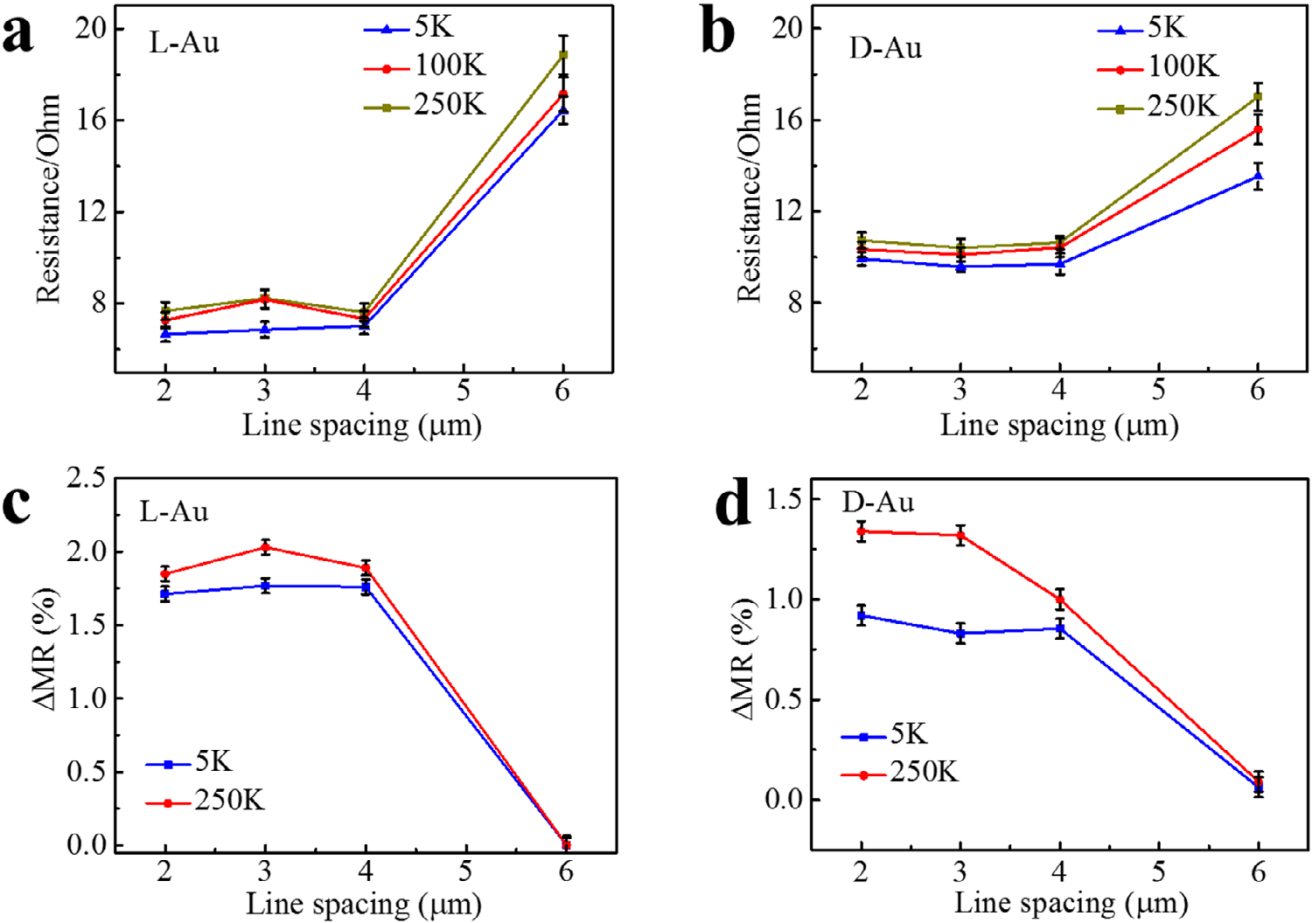
Distance dependence of the transport and magnetoresistance. The transport properties of the chiral gold as a function of distance between the electrodes. a,b) The resistance as a function of distance for L and D films respectively. c,d) The ΔMR (%) =|MR (%)|_−1T_ +  |MR (%)|_+ 1T_ where MR is the magneto resistance measured with a magnetic field, B, of either −1 T or +1 T. MR = [I(±B)‐I_0_]/I_0_ where I(±B) is the current in the presence of the magnet which is pointing either parallel (+) or antiparallel (−) relative to the current direction and I_0_ is current with no magnet.

**Figure** **2** presents the transport properties of the chiral film as a function of the distance between the two inner electrodes in the device. The resistance is presented in Figure 2a,b for L and D chiral gold, respectively. While for distances of up to 4 µm the conduction increases weakly as a function of distance, for larger distances the resistance increases very significantly and reaches the value obtained with achiral gold film (see Figure S2, Supporting Information). Within the signal‐to‐noise ratio of the experiments, the resistance exhibits almost no temperature dependence.

To relate this long‐range low resistance transport to spin, ΔMR (%) =|MR (%)|_−1T_ +  |MR (%)|_+ 1T_ was measured, where MR is the magneto resistance with a magnetic field, B, of either −1T or +1T. MR = [I(±B)−I_0_]/I_0_ as I(±B) is the current in the presence of the magnet which is either pointing parallel (+) or antiparallel (−) relative to the current direction and I_0_ is current with no magnet (Figure 2c,d). ΔMR is an indicator of the spin selectivity in conduction. While for distances of up to 4 µm, this value remains about constant, for longer distances it decays, and it is practically zero at 6 µm. Please note that the values of ΔMR are consistently larger for the L gold as compared to the D gold. This is consistent with the intensity of the CD spectra, and it is probably a result of the D tartaric acid being less enantiopure than the L enantiomer. For short distances, it seems that ΔMR increases somewhat with temperature. At these distances, the contact resistance is dominant, and therefore the total resistance does not depend on the length up to ≈4 µm. To verify the effect of the spin, we measured the dependence of the MR on the angle between the current and the magnetization direction of the magnetic electrode. The results are shown in Figures S3 and S4 (Supporting Information) and are consistent with the cosine square dependence previously observed for the CISS effect.^[^ ^22^ ^]^ The complete details of the MR measurements are shown in Figure S5 (Supporting Information).

**Figure 3:**
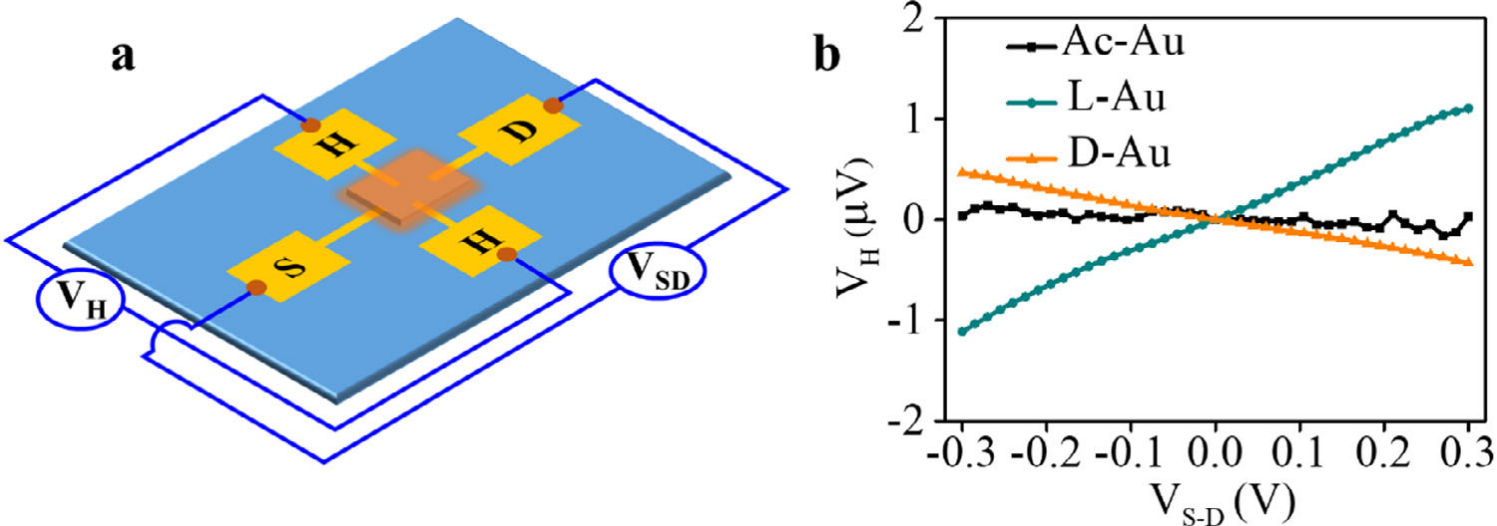
The chirality induced the Hall signal. a) The Hall device used in this experiment, b) The Hall signals from the device where the distance between the source (S) and drain (D) electrodes is 4 µm. The slope of the Hall signal dependent on the voltage is positive for the L‐Au (red) and negative for the D‐Au (blue). In the case of achiral gold (Ac–Au) there is no significant signal.

For the chiral gold, we obtained a Hall signal, as shown in **Figure** **3**. Here, no external magnetic field was applied (Figure 3b). The explanation for this Hall signal will be discussed below. The Hall signal is presented as a function of the voltage applied between the source and drain electrodes and the plots for the L and D gold have opposite signs, as expected. As in the MR signal, the slope value for the L gold is larger than for the D gold, indicating higher enantiopurity of the L enantiomer. For achiral gold, no signal is observed.

The frequency dependence of the Hall signal was measured up to a frequency of 1 GHz. Longitudinal AC currents were applied, starting at 1 MHz and increasing to 1 GHz in 10 MHz increments. At each frequency, the transverse DC Hall voltage was measured. This DC Hall voltage arises due to an asymmetry in the number of electrons at the edges of our device when the current frequency is smaller than the inverse of the spin‐lifetime. More details of the origin of the DC Hall voltage are given in the Supporting Information. To avoid interference from signals related to the change in frequency, there is a 60 s delay between each frequency change and the measurement of the Hall voltage. High‐frequency coaxial cables were used, and the sample was enclosed within an aluminum box to prevent external noise during the measurements. More details are provided in the Supporting Information.

**Figure 4:**
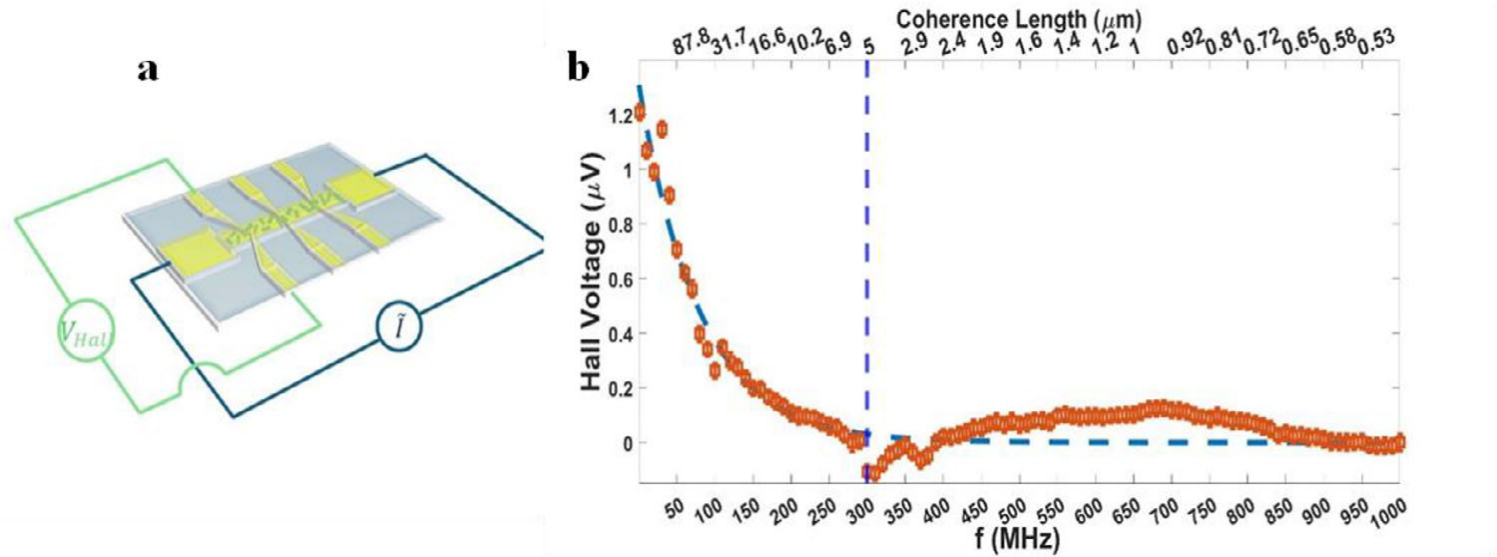
The chirality induced frequency dependent Hall voltage. a) The Hall device used in the experiment. The distance between the current source and drain is 4 mm, and the width of the Hall bar is 100 µm. b) The frequency‐dependent Hall voltage and the corresponding coherence length (upper scale). The different frequency‐dependent behavior regimes agree with different transport regimes.

The frequency‐dependent measurements, shown in **Figure** **4**, reveal a decrease in the Hall signal with increasing frequency showing a half lifetime of ≈160 MHz that corresponds to a “spin transport distance” of ≈5 µm in the gold. The term “transport distance” must be clarified in the context of a chiral conductor, since it is not related directly to the spin‐free mean path in the material. Rather, it is the net distance that the electrons cross in one current half cycle. This was calculated using the drift velocity, which is found using the measured spin‐lifetime. The full calculation of the coherence length is shown in the Supporting Information. When spin‐polarized electrons are injected into a chiral material two processes occur. The first is that the spin polarization decays due to collisions, where the decay constant is *k_D_*, and the second is the buildup of spin polarization due to the CISS effect with a buildup constant k_B_. Hence, the spin concentration, S, at any time is given by:

(1) $$S=Wexp\left[-\;\left(k_{D}-k_{B}\right)t\right]$$

where *W* is the Lambert function.^[^ ^23^ ^]^ Assuming that the Hall signal is proportional to the value of the spin polarization in the current, we can derive *k_D_−k_B_*_._ The details on the calculation of the transport distance are given in the Supporting Information.

As a result of the competing effects, spin polarization simultaneously decays and builds up, there is an “effective lifetime” of the spin population that corresponds to a frequency of 160 MHz, which coincides with the spins’ path between 4 and 5 µm, as was observed in Figure 2. The fit to Equation (1) results in (*k_D_* − *k_B_*) ≈ 1.6 × 10^8^ s^−1^. This result means that the spin equilibrium time within the chiral gold is ≈1 ns, much longer than in achiral multi‐crystalline gold, where the lifetime is on the order of 10 ps.^[^ ^24^ ^]^ This lengthening of the lifetime is also a result of coupling between the electron's linear momentum and its spin in chiral materials, which “protect” the spin and reduce backscattering.^[^ ^25^ ^]^ Note that the fit reveals the average behavior but distinct transport regimes can be identified at a closer look.

### 2.1 Theoretical Modeling

**Figure 5:**
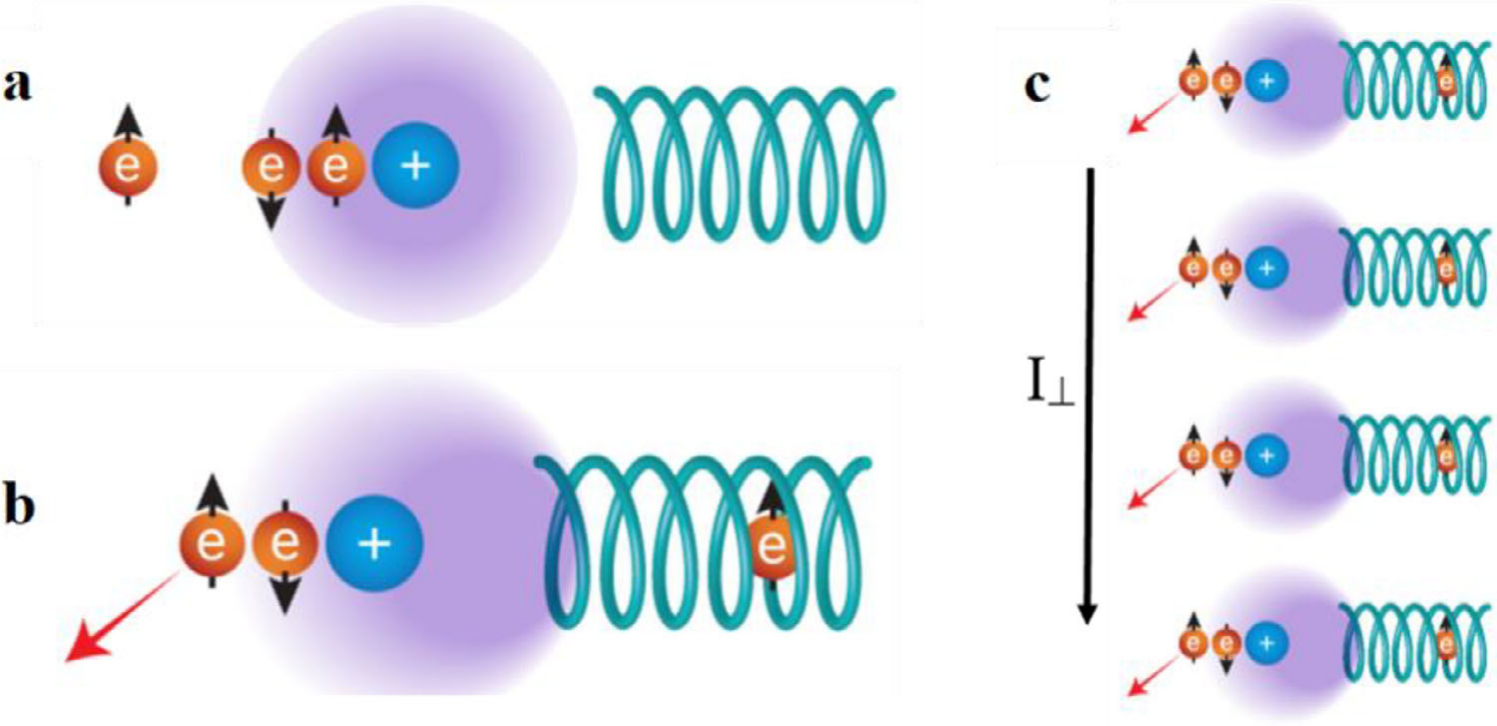
A scheme of the topological anomalous Hall effect (TAHE). a) Electrons moving in the chiral system are spin‐polarized due to the CISS effect. b) When the moving electron collides with an atom, it polarizes the charge on the atom so that some charge moves into the nearby atoms that are in a chiral system. Due to the chirality, this displaced charge is spin‐polarized, leaving behind (partial) electrons with specific spin. Hence, the effective spin–orbit coupling has two components, the relativistic effect of the electric field (common SOC) and spin exchange interaction. Therefore, the moving electron is scattered non isotropically, to one specific side, depending on the interaction. c) In the case of bulk materials, the scattering results in charge motion perpendicular to the original current, I_⊥_, creating the Hall potential (see text).

Regarding the Hall signal, the question is how it exists without stationary unpaired electrons, hence polarized spins, in the material. **Figure** **5** presents schematically the mechanism of what we refer to as *topologically anomalous Hall* (TAH) effect. The detailed theory is provided below and in the Supporting Information. The transport of electrons through a chiral system is spin dependent due to the CISS effect. Hence the transported electrons are spin‐polarized. When spin‐polarized electrons collide with an atom embedded in a chiral system, an electron that was residing on the atom, or part of it, is repelled to the neighboring atoms. Because this charge is moving in a chiral potential, the repelled electron has a specific spin, leaving an excess of the opposite spin behind to interact with the colliding spin‐polarized electron. Hence, the transmitted spin‐polarized electrons always collide with electrons having mainly one specific spin via spin exchange interaction (see Figure 5). This spin exchange interaction and the spin–orbit coupling are responsible for the electrons being scattered in an anisotropic manner, creating the Hall potential. The anisotropy in spin scattering is similar to that obtained in Rashba^[^ ^26^ ^]^ and Mott^[^ ^27^ ^]^ scattering and is a result of the breaking of the symmetry when an electron in a specific spin state is scattered from a system having spin–orbit coupling.

Hence, the TAH effect has two stages i) Spin selective polarization of the atoms by the transferred electrons, causing a transient spin alignment in the atom that interacts with the colliding electron. ii) Anisotropic scattering of the transmitted electrons from the atoms.

It was shown before that indeed charge polarization in a chiral medium is accompanied by transient spin polarization. This transient spin polarization is responsible for the TAHE.^[^ ^15^ ^]^ The model calculations presented below provide detailed insight into the mechanism.

The model is based on describing the chiral metal in which two bands are coupled by phonons‐assisted spin–orbit coupling and spin exchange interactions.^[^ ^28^, ^29^ ^]^ Without going deeply into details, which are presented in the SI, the properties of the model structure crucially depend on two aspects, i) phonon‐assisted coupling of the bands which is responsible for making the metal chiral, and ii) phonon‐assisted inter‐band spin–orbit coupling. Couplings via phonons are essential since they provide polarizability to the electronic structure, which is important for the transport properties discussed next.

In the context of transport properties pertaining to the Hall effect, the phonon‐assisted band coupling is effectively parametrized by Δ  =  (Δ_*x*_,Δ_*y*_,Δ_*z*_), where Δ_*x*_ and Δ_*y*_ denote the transverse coupling components and Δ_*z*_ the longitudinal component. The former coupling splits the degeneracy of the two bands by reorganizing the electronic density into chiral conformation with opposite chirality. However, this coupling does not break the energetic degeneracy of the bands, which the longitudinal component Δ_*z*_ does. By requiring that Δ breaks inversion symmetry, we define chiral symmetry breaking whenever the two chiral bands are non‐degenerate.

The phonon‐assisted inter‐band spin–orbit coupling is parametrized in terms γ_*i*_, *i*  =  *x*, *y*, *z*, all which are all three component vectors. The transverse couplings γ_*x*_ and γ_*y*_ generating spin‐texture in the electronic structure, whereas γ_*z*_ leads to the spin‐polarization of the two bands canceling whenever these are degenerate. It should be stressed that γ_*i*_ has to break inversion symmetry to form a viable source of spin–orbit coupling.

The transverse currents are given by

(2) $$\left(\begin{array}{c} J_{x}\\ J_{y} \end{array}\right)=-\frac{eh}{m_{e}}\gamma_{z}\sqrt{\gamma_{x}^{2}+\gamma_{y}^{2}}\sqrt{\Delta_{x}^{2}+\Delta_{y}^{2}}I_{J}\left(E\right)\left(\begin{array}{c} \cos\delta \\ \sin\delta \end{array}\right)$$

here, the externally applied electric field *E*, is included in the integral *I_J_* (*E*), whereas the angle δ, tanδ  = Δ_*y*_ /Δ_*x*_, is related to the spatial anisotropy of the chirality.

It is necessary that the degeneracy of the two chiral bands (chiral symmetry) is broken since this leads to different energies and hence to spin polarization namely, there is an occupation imbalance between the bands. Because of the occupation imbalance, the majority band contributes more to the current, and since this band has a well‐defined chirality, the electrons’ spins are aligned according to this chirality and thereby forced to bend into a specific direction into the transverse direction. The occupation imbalance between the bands can be interpreted as the electrons acquiring a directional preference in their scattering of the phonons, as demonstrated in Figure 5B.

The broken chiral degeneracy between the chiral bands leads to an induced effective magnetic moment. Under the same condition as above, the transverse components of this moment, pertaining to the anomalous Hall effect, can be expressed as

(3) $$\left(\begin{array}{c} M_{x}\\ M_{y} \end{array}\right)=\sqrt{\gamma_{x}^{2}+\gamma_{y}^{2}}\;\sqrt{\Delta_{x}^{2}+\Delta_{y}^{2}}\Delta_{z}I_{M}\left(E\right)\left(\begin{array}{c} \cos\delta \\ \sin\delta \end{array}\right)$$

The presence of both γ_⊥_ and Δ_⊥_ indicates that the same mechanisms, chirality, and spin–orbit coupling, are responsible for the induced transverse moment as well as for inducing the spin polarization in the current. In addition, the broken degeneracy between the chiral bands is presented here explicitly through the parameter Δ_*z*_.

The spin exchange interaction, which plays an important role in the ATH effect, is a result of the electronic polarizability of the system due to electro‐phonon interactions. The theoretical model indicates that indeed the electrons that are transported through the film induce charge polarization in the atoms. Due to the chirality of the system, this charge polarization is spin dependent and as a result, the polarized atoms have at least partial spin polarization (see Figure 5). This spin interacts with the moving electrons and causes anisotropic scattering that is expressed as a Hall signal. Hence, the ATH effect is a result of induced charge polarization in the chiral material which creates transient spin polarization on the atoms. The phonons contribute to the charge polarization and therefore the effect is efficient also at room temperature. The Hall effect observed is a result of unpaired electrons like in the common anomalous Hall effect. However, unlike in the common anomalous effect, our system does not contain unpaired electrons. Hence, the unpaired spins are the result of charge scattering in the chiral system, a topological effect.

## 3 Conclusion

Here two important findings were explored. The first is the long‐range spin transport in chiral metal at room temperature and the second is the anomalous topological Hall effect. The magneto resistance results (Figure 2) show a weak temperature dependence, where the signal increases slightly with temperature. This observation is consistent with a weak coupling of the electrons with phonons, however, it is evident that phonons assist the CISS effect, as was indicated in former works.^[^ ^22^ ^]^ Therefore, the results presented here are due to the coupling between the transmitted electrons and the nuclei's degree of freedom, as presented in the model. It is a dynamical spin polarization effect.

The spin dependent electrons‐phonons interaction is a result of the anisotropic polarizability of the system which is the manifestation of the chirality. The results indicate that chiral metals may serve as low‐resistance interconnects in microelectronics as well as spin dependent interconnects in spintronics.

## Conflict of Interest

The authors declare no conflict of interest.

## Supporting information



Supporting Information

## Data Availability

The data that support the findings of this study are available from the corresponding author upon reasonable request.
