# Supplementary material for: Long‐Range Spin Transport in Chiral Gold
Source: Adv Mater. 2025 Jun 11;37(34):2506523. doi: 10.1002/adma.202506523 (PMC12392861; doi:10.1002/adma.202506523)
Supplement: Supplementary file 1 — Supporting Information [file ADMA-37-2506523-s001.docx]

**Long-Range Spin Transport in Chiral Gold**

Tapan Kumar Das^1a^, Offek Marelly^2a^, Shira Yochelis,^2^ Yossi Paltiel^2^, Ron Naaman^1^, and Jonas Fransson^3^

1. Department of Chemical and Biological Physics, Weizmann Institute, Rehovot 76100, Israel
2. Applied Physics Institute, Center for Nanoscience and Nanotechnology, Hebrew University of Jerusalem, Jerusalem 91904, Israel
3. Department of Physics and Astronomy, Uppsala University, Uppsala 752 21, Sweden

**Materials, experimental details and the device fabrication:**

**Device fabrication and measurements:** A four electrode planer device was produced on Si (100)/SiO_2_ substrate. The two outer electrodes were made from Au (60 nm) on top of Ti (8 nm) as adhesive layer. A constant current was applied between them. Other two inner electrodes were deposited between them, one also from Ti (8 nm)/Au (60 nm) and the other Ti (8 nm)/Ni (60 nm)/Au (10nm). The voltage was measured between them. All the metals were deposited by E-beam evaporator. Transport and magnetoresistance (MR) measurements were performed using a cryogenic system (Cryogenics, Ltd.), with a magnetic field of up to ±1 T applied at various angles relative to the current and at various temperatures. The MR was measured using a constant current of 0.001 A with a Keithley 2400 current source, while the voltage was recorded using a Keithley 2182A nanovoltmeter.

**Chiral gold deposition**: Electrochemical depositions have been performed on the four electrodes device that served as the working electrode (WE) in a configuration shown in Figure S1. Prior to chiral gold deposition, the substrate was cleaned in boiling acetone followed by ethanol, each for 10 min. The Au layers were electrochemically deposited using a solution of Na_3_[Au(S2O_3_)_2_]·H_2_O, Na_2_S_2_O_3_, Na_2_SO_3_, and chiral tartaric acid in water at pH 6.5. The layer was grown Chronovoltammetry (CV) and Chrono Chronoamperometry (CA) on the flat surface at a constant voltage of −0.9 V using Pt as the counter electrode (CE) and Ag/AgCl/KCl_sat_, is the reference (RE).

**Circular Dichroism (CD) Spectroscopy:** The Circular Dichroism spectroscopy measurements were performed using a Chirascan spectrometer with a thermoelectrically controlled single cell holder. For the CD measurement, the chiral Au was deposited on ITO substrate.


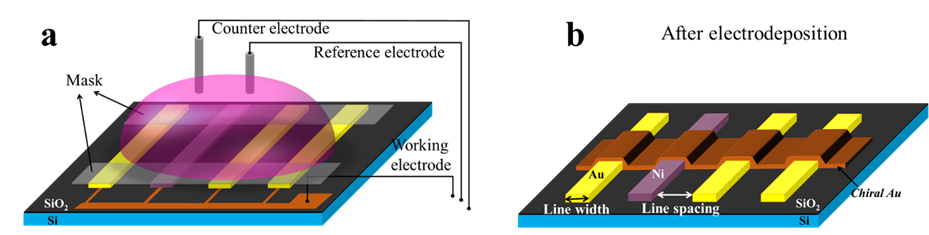


**Fig. S1: Device fabrication:** a) Schematic of the experimental setup used for electrochemical deposition of Chiral Au. All the four electrodes are connected in parallel and served as the working electrode. A Pt wire was used as the counter electrode (CE), Ag/AgCl/KCl_sat_, is the reference electrode (RE). The materials details and conditions for the deposition are given in the text. b) The device after the deposition of the chiral Au.

**Results**

**Temperature and angular dependent Magnetoresistance measurement:**

Magnetoresistance (MR) was measured for the chiral gold coated on the four electrode planer device. The input current of 0.001A was applied between Ni and gold (two inner electrodes) and the voltage was measured across the two outer electrodes as shown in Figure S2b. The MR was measured in an in-plane magnetic field as function of temperature shown in Figure S2b and 2c. The direction of the magnetic field was varied from in-plane to out of plane while keeping the direction of current fixed. The normal resistance was measured as a function of the distance between the two inner electrodes, for chiral-Au and achiral Au. The results are shown in Fig. S2. The measured MR is defined as $MR (\%)=\frac{R\left( B \right)-R(0)}{R(0)}\times100$, where R(B) and R(0) are the in-field and zero-field resistances respectively. The spectra were collected by scanning the magnetic field between -1.0 and 1.0 T at different angles and temperatures.


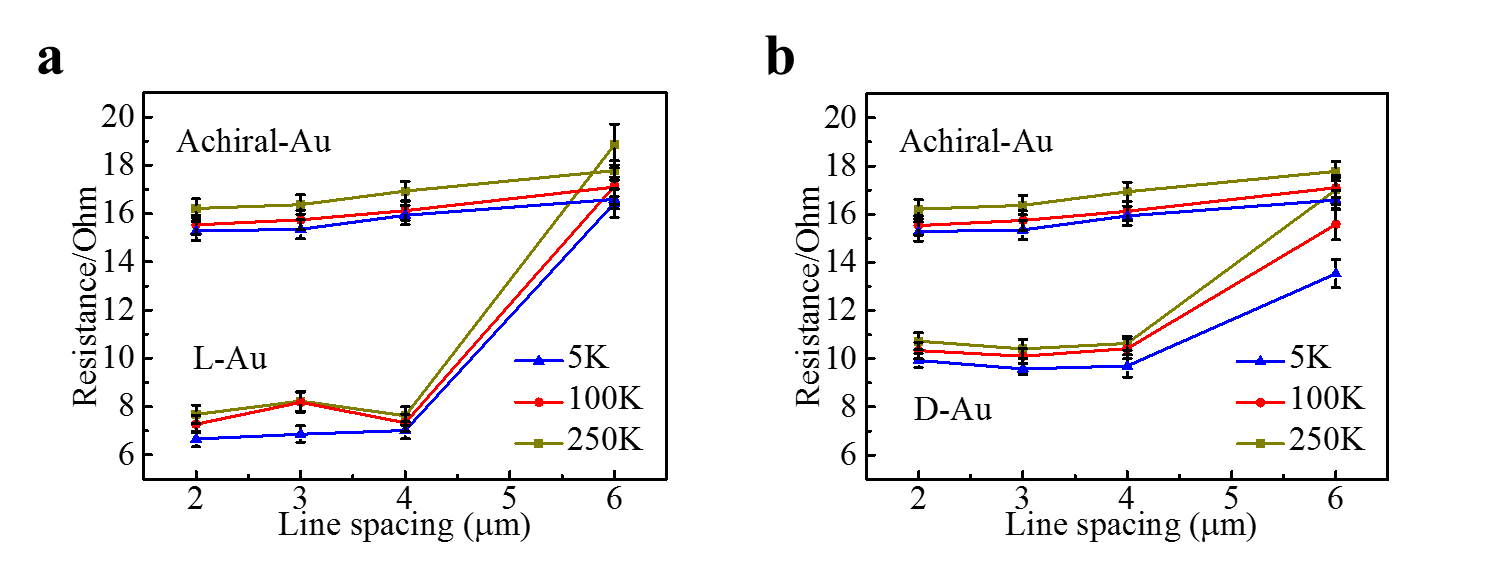


**Fig. S2:** The transport properties of the chiral and achiral gold as a function of temperature and the distance between the electrodes.


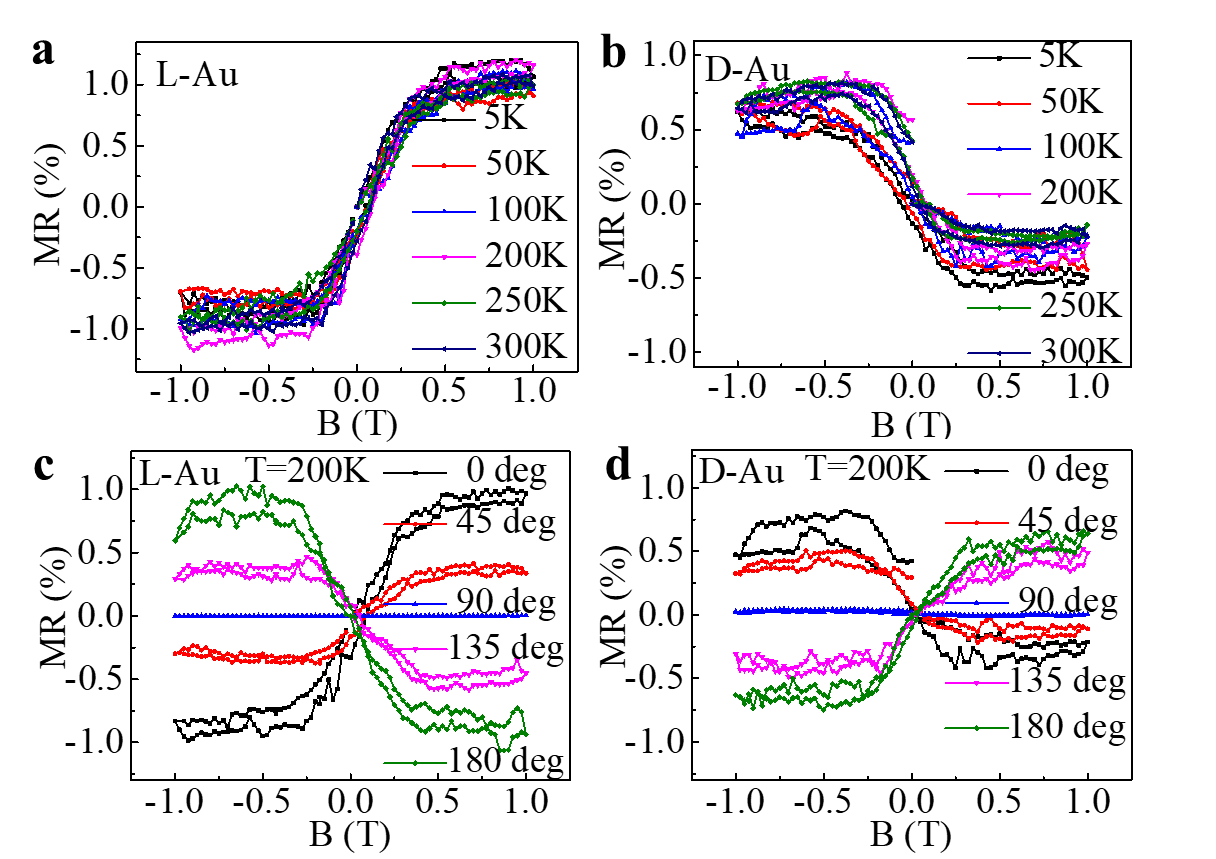


**Fig. S3:** **Magnetoresistance and its angular variation:** The variation of MR as a function of magnetic field at different temperature, a) for L-Au and b) for D-Au. The angular variation of magnetoresistance as a function of magnetic field at temperature 200K for L-Au and D-Au are shown in figure c) and d) respectively. A current of I=0.001A was applied in all the measurements and the magnetic field, B, was applied at different angles with the direction of current. The measurements were done on a device with 4 μm line spacing and 4 μm line width.

**
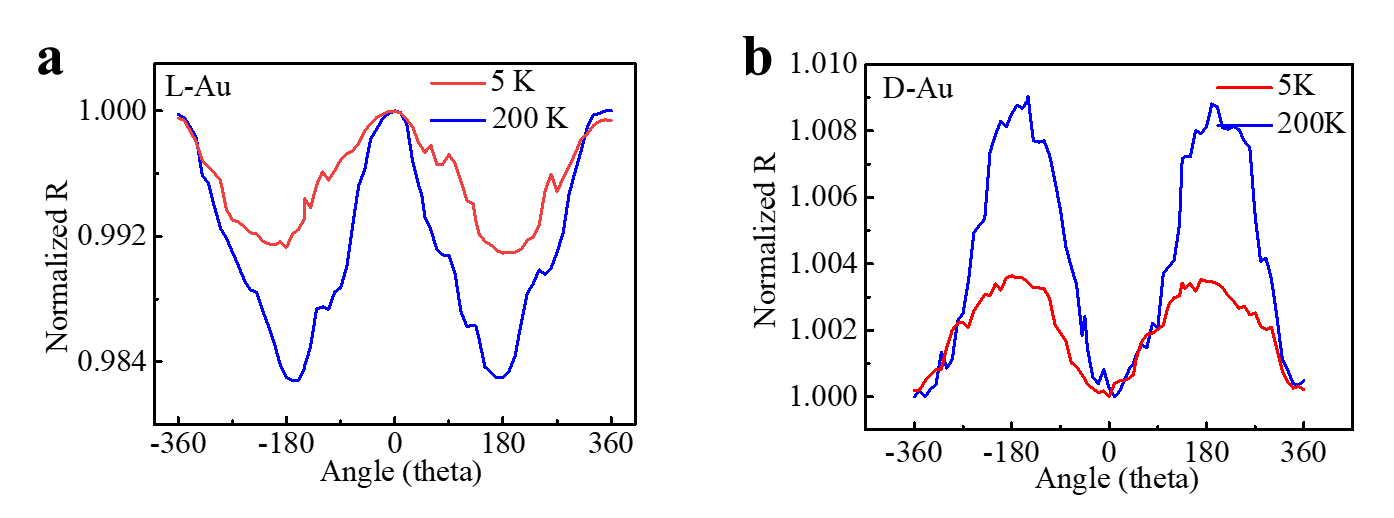
**

**Fig. S4: Magnetoresistance and its angular variation:** The angular variation of MR as a function of the angle between the direction of current and magnetic field, a) for L-Au and b) for D-Au. The magnetoresistance was measured at temperature of 5K and 200K for both samples. A current of I=0.001A and the magnetic field of +1T were applied for all the measurements.


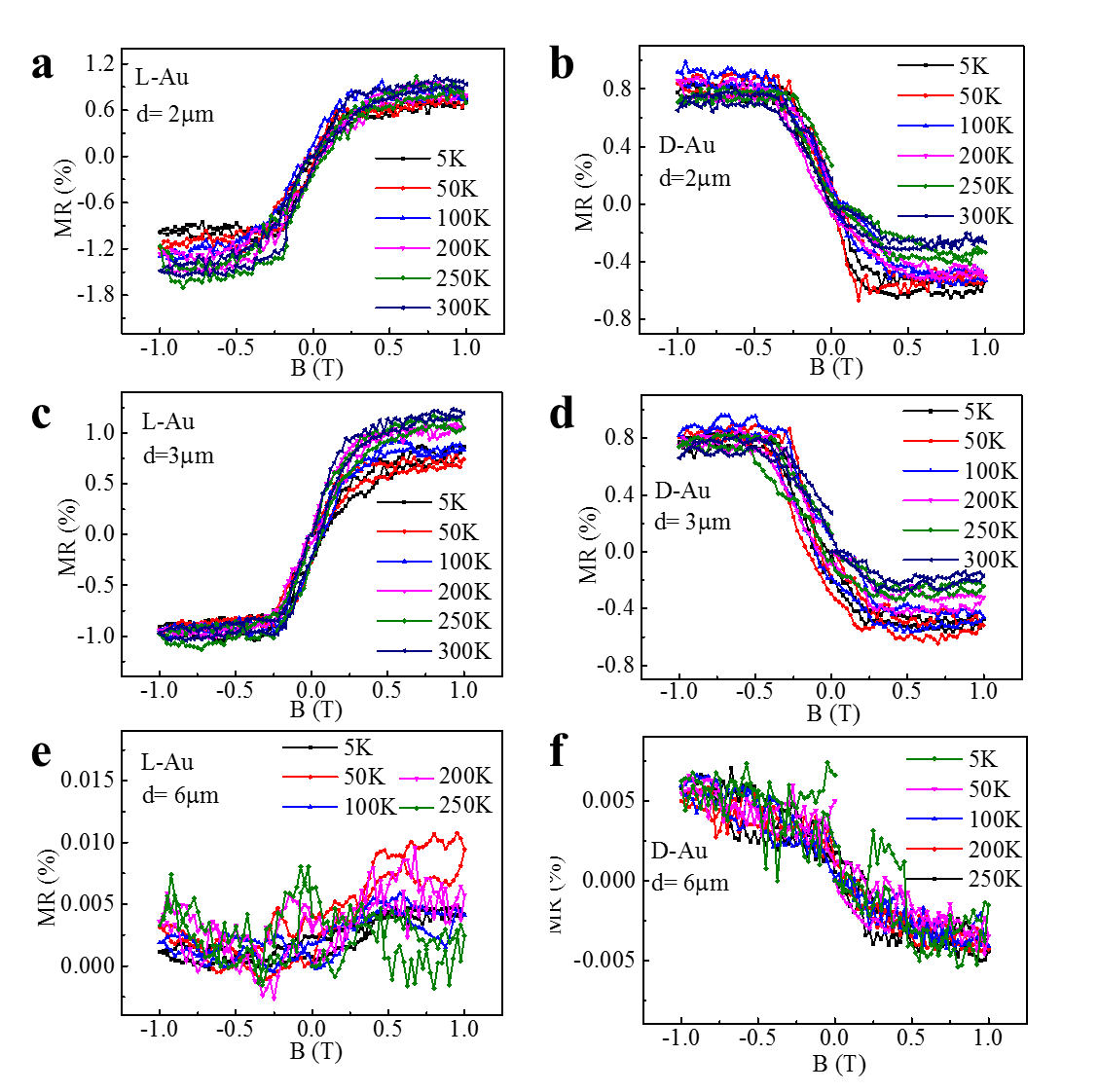


**Fig. S5: Temperature and line spacing dependent Magnetoresistance:** The variation of MR as a function of magnetic field at different temperatures, for the line spacing of d=2 µm for A) L-Au and B) for D-Au, for the line spacing of d=3 µm for C) L-Au and D) for D-Au and 6 µm for E) L-Au and F) for D-Au respectively. A current of I=0.001A was applied for both samples. These measurements were done on a device with 4 µm line width.

**Frequency Dependent Hall signal measurements**

To determine the spin lifetime, we measured the Hall voltage in the chiral gold as a function of the frequency of the AC potential. It is expected that once the frequency of the current is higher than the inverse of the spin lifetime, the electrons will maintain their spin polarity throughout the whole current cycle. The number of electrons on the edges of the chiral gold will be equal – the net Hall voltage will be zero. *If the electrons do not maintain their spin polarity throughout the cycle, some of the electrons will move from one side of chiral gold to the other when the current changes direction. The unequal number of electrons on the edges gives rise to a nonzero net Hall voltage.* By measuring the Hall voltage as a function of the voltage/current frequency, it is possible to determine the effective lifetime of the spin. The Hall device (see Figure S6) was fabricated by thermal evaporation and was then coated with chiral gold using electrodeposition. Longitudinal AC currents, starting at 1MHz and increasing to 1GHz in 10MHz increments, were driven through the device using a Keysight N15171 signal generator. At each frequency, the transverse DC Hall voltage was measured using a Keithley 2182A Nanovoltmeter. To avoid interference from signals related to the change in frequency, there is a sixty second delay between each frequency change and the measurement of the Hall voltage. To eliminate voltage arising from misalignment of the voltage terminals, the voltage of the achiral sample was subtracted from the voltage of the chiral one. High frequency coaxial cables were used, and the sample was enclosed within an aluminum box to prevent external noise during the measurements.


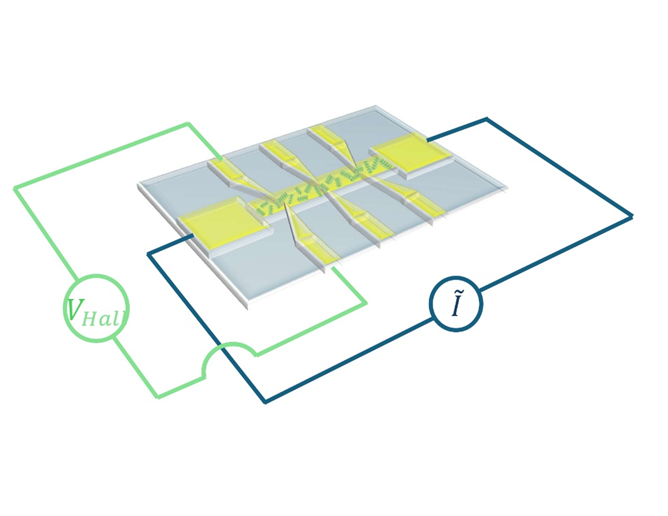


**Fig. S6: A scheme of the high frequency Hall effect measurement system.**

In Figure 4 the time was obtained from the frequency and was fitted to equation 1. To determine the average distance over which the electrons’ spin polarization is maintained, the drift velocity of the electrons was calculated. Using the drift velocity, it is possible to calculate the distance the electrons cross in one current cycle. It is important to note that this is the net distance that the electrons cross in the direction of the current, and not the mean free path of the electrons as they move inside the chiral gold.

The drift velocity is calculated using the Drude model:

$$v_{drift}=\frac{e\cdot V_{RMS}\cdot\tau}{l\cdot m^{*}}$$

where $e$ is the electron charge, $l=1400 \mu m$ is the distance between the voltage taps, $m^{*}$ is the effective mass of the electron, and $V_{RMS}$ is the RMS longitudinal voltage applied on the device. In our experiments, $V_{RMS}\approx10mV$.

The lifetime $\tau$ is derived from both the spin lifetime, as well as the period of the driving current. This is because both, decay of the spin polarity and electron scattering must be accounted for. Then lifetime is taken as:

$$\frac{1}{\tau}= \frac{1}{\tau_{spin-lifetime}}+\frac{1}{\tau_{current}}$$

where $\tau_{spin-lifetime}\approx4.6 ns$, is the spin lifetime derived from the experiment, and $\tau_{current}$ is the period of the current. Once the drift velocity at a given current frequency is found, the coherence length is calculated as the product of the drift velocity and the period of the current:

$$l_{coherence}=v_{drift}\cdot\tau_{current}$$

**Theoretical Model**

An effective model describing a chiral metal with spin-orbit coupling can be formulated through the Hamiltonian $\mathcal{H=}\underset{\boldsymbol{k}}{\sum}\Psi_{\boldsymbol{k}}^{\dagger}H\Psi_{\boldsymbol{k}}$, where the four component spinor $\Psi_{\boldsymbol{k}}=(\psi_{1\boldsymbol{k}} \psi_{2\boldsymbol{k}})^{t}$ is provided in terms of the two-component spinor $\psi_{\mu\boldsymbol{k}}=(\psi_{\mu\boldsymbol{k}\uparrow} \psi_{\mu\boldsymbol{k}\downarrow})^{t}$ for the band $\mu=1,2$. Furthermore, $H$ is a $4\times4$-matrix defined by

$H=\left( \begin{matrix} \boldsymbol{\epsilon}_{1\boldsymbol{k}} & (\Delta_{x\boldsymbol{k}}-i\Delta_{y\boldsymbol{k}})\sigma^{0}+(\gamma_{x\boldsymbol{k}}-i\gamma_{y\boldsymbol{k}})\cdot\boldsymbol{\sigma} \\ (\Delta_{x\boldsymbol{k}}+i\Delta_{y\boldsymbol{k}})\sigma^{0}+(\gamma_{x\boldsymbol{k}}+i\gamma_{y\boldsymbol{k}})\cdot\boldsymbol{\sigma} & \boldsymbol{\epsilon}_{2\boldsymbol{k}} \end{matrix} \right)$,

where each entry itself is a $2\times2$-matrix, Here, the intraband energy spectra are defined by the matrices $\boldsymbol{\epsilon}_{\mu\boldsymbol{k}}=[\varepsilon_{\boldsymbol{k}}+(-1)^{\mu}\Delta_{z\boldsymbol{k}}]\sigma^{0}+[\boldsymbol{\alpha}_{\boldsymbol{k}}+(-1)^{\mu}\gamma_{z\boldsymbol{k}}]\cdot\boldsymbol{\sigma}$, where $\varepsilon_{\boldsymbol{k}}$ is an energy dispersion, $\boldsymbol{\alpha}_{\boldsymbol{k}}$ denotes the elastic intraband spin-orbit coupling, whereas $\boldsymbol{\Delta}_{\boldsymbol{k}}=(\Delta_{x\boldsymbol{k}},\Delta_{y\boldsymbol{k}},\Delta_{z\boldsymbol{k}})$ is the interband coupling which introduces chirality in the band structure. Furthermore, an interband spin-orbit coupling is provided by $\boldsymbol{\gamma}_{i\boldsymbol{k}}$, where $\boldsymbol{\gamma}_{i\boldsymbol{k}}$, $i=x,y,z$, are three component vectors. The couplings $\boldsymbol{\Delta}_{\boldsymbol{k}}$ and $\gamma_{i\boldsymbol{k}}$ are defined below. It should be noticed, however, that both $\boldsymbol{\Delta}_{\boldsymbol{k}}$ and $\boldsymbol{\gamma}_{i\boldsymbol{k}}$ are required to be odd (or at least not even) under inversion symmetry, such that $\boldsymbol{\Delta}_{\boldsymbol{k}}$ accounts for chirality and $\boldsymbol{\gamma}_{i\boldsymbol{k}}$ for a spin-orbit coupling.

Considering the transport properties of a material with these properties, the current can be formulated as

$\boldsymbol{J}(\boldsymbol{r})=-\frac{eh}{m_{e}}\int\frac{\partial f_{0}}{\partial\omega}g(\omega)K_{\boldsymbol{k}}(\omega)\boldsymbol{k}\frac{d\boldsymbol{k}}{\Omega}\frac{d\omega}{2\pi}$, (1a)

$K_{\boldsymbol{k}}(\omega)=2Im\underset{\mu}{\sum}\frac{\omega-\varepsilon_{\mu\boldsymbol{k}}-\Sigma_{c\mu}(\omega;\boldsymbol{k})}{[\omega-\varepsilon_{\mu\boldsymbol{k}}-\Sigma_{c\mu}(\omega;\boldsymbol{k})]^{2}-[\boldsymbol{\alpha}_{\mu\boldsymbol{k}}+\boldsymbol{\Sigma}_{s\mu}(\omega;\boldsymbol{k})]^{2}}$, (1b)

where $K_{\boldsymbol{k}}\left( \omega\right)$ and *g*(*w*) are proportional to the electron spectral density and to the external electric field, respectively, whereas $\partial f_{0}/\partial\omega$ is the derivative of the Fermi function. Here, the $\varepsilon_{\mu\boldsymbol{k}}=\varepsilon_{\boldsymbol{k}}+(-1)^{\mu}\Delta_{z\boldsymbol{k}}$ and $\boldsymbol{\alpha}_{\mu\boldsymbol{k}}=\boldsymbol{\alpha}_{\boldsymbol{k}}+(-1)^{\mu}\gamma_{z\boldsymbol{k}}$, whereas the self-energies $\Sigma_{c\mu}$ and $\boldsymbol{\Sigma}_{s\mu}$ are defined by

$\Sigma_{c1}(\omega;\boldsymbol{k})=\frac{(\Delta_{\perp\boldsymbol{k}}^{2}+\gamma_{\perp\boldsymbol{k}}^{2})(\omega-\varepsilon_{2\boldsymbol{k}})+2\alpha_{2\boldsymbol{k}}\gamma_{\perp\boldsymbol{k}}\Delta_{\perp\boldsymbol{k}}cos\phi_{\boldsymbol{k}}}{[\omega-E_{2+}(\boldsymbol{k})][\omega-E_{2-}(\boldsymbol{k})]}$, (2a)

$\boldsymbol{\Sigma}_{s1}(\omega;\boldsymbol{k})=\frac{2\gamma_{\perp\boldsymbol{k}}\Delta_{\perp\boldsymbol{k}}(\omega-\varepsilon_{2\boldsymbol{k}})cos\phi_{\boldsymbol{k}}+\alpha_{2\boldsymbol{k}}(\Delta_{\perp\boldsymbol{k}}^{2}+\gamma_{\perp\boldsymbol{k}}^{2})}{[\omega-E_{2+}(\boldsymbol{k})][\omega-E_{2-}(\boldsymbol{k})]}$, (2b)

and where $\Sigma_{c2}$ and $\boldsymbol{\Sigma}_{s2}$ are obtained by interchanging the subscripts 1 and 2. The anisotropy is seen through the **k**- and angle-dependence. The subscripts *c* and *s* refer to that the corresponding self-energy is related to charge and spin degrees of freedom, respectively. In the expressions for the self-energy, the quantity $\Delta_{\perp\boldsymbol{k}}^{2}=\Delta_{x\boldsymbol{k}}^{2}+\Delta_{y\boldsymbol{k}}^{2}$ and $\tan\phi_{\boldsymbol{k}}=\Delta_{y\boldsymbol{k}}/\Delta_{x\boldsymbol{k}}$ define the chirality, $\gamma_{\perp\boldsymbol{k}}^{2}=\gamma_{x\boldsymbol{k}}^{2}+\gamma_{y\boldsymbol{k}}^{2}$, $\gamma_{i\boldsymbol{k}}=|\boldsymbol{\gamma}_{i\boldsymbol{k}}|$, the interband spin-flip scattering processes, whereas $E_{\mu\pm}(\boldsymbol{k})=\varepsilon_{\mu\boldsymbol{k}}+s|\boldsymbol{\alpha}_{\mu\boldsymbol{k}}|$ is the modified energy dispersion due to the interband spin-orbit coupling.

It is crucial to notice that both chirality and interband spin-orbit coupling have to be present in the structure for the Hall effect to emerge. This can be seen in the numerator of the kernel $K_{\boldsymbol{k}}$, specifically in $\Sigma_{c\mu}(\omega;\boldsymbol{k})$, in which the product $\alpha_{\mu\boldsymbol{k}}\gamma_{\perp\boldsymbol{k}}\Delta_{\perp\boldsymbol{k}}\cos\phi_{\boldsymbol{k}}$ signifies these conditions. Moreover, since $\boldsymbol{\alpha}_{\mu\boldsymbol{k}}=\boldsymbol{\alpha}_{\boldsymbol{k}}+(-1)^{\mu}\boldsymbol{\gamma}_{z\boldsymbol{k}}$, it is clear that a non-zero intraband spin-orbit coupling $\boldsymbol{\alpha}_{\boldsymbol{k}}$ is not a necessary condition for the Hall effect to emerge, however, it may both strengthen and weaken the effect, depending on the relation between $\boldsymbol{\alpha}_{\boldsymbol{k}}$ and $\boldsymbol{\gamma}_{z\boldsymbol{k}}$. It is however necessary that the degeneracy of the two chiral bands (chiral symmetry) is broken, which is indicated by the vector $\boldsymbol{\gamma}_{z\boldsymbol{k}}$, since this leads to different energies corresponding to the products $\alpha_{\mu\boldsymbol{k}}\gamma_{\perp\boldsymbol{k}}\Delta_{\perp\boldsymbol{k}}\cos\phi_{\boldsymbol{k}}$, for $\mu=1,2$.

A simplified expression for the current can be obtained by defining the angle $\varphi-\delta$ through $\tan(\varphi-\delta)=\Delta_{y\boldsymbol{k}}/\Delta_{x\boldsymbol{k}}$, where $\varphi$ denotes the azimuthal variation of $\boldsymbol{k}$, and by suppressing the $\boldsymbol{k}$-dependence of $\Delta_{\perp\boldsymbol{k}}$ and $\boldsymbol{\gamma}_{i\boldsymbol{k}}$, $i=x,y,z$, as well as omitting the angular variations of the denominator of $K_{\boldsymbol{k}}$. Then, the traverse currents are given by

$\left( \begin{aligned} J_{x} \\ J_{y} \end{aligned} \right)=-\frac{eh}{m_{e}}\gamma_{z}\gamma_{\perp}\Delta_{\perp}I_{J}(\boldsymbol{E})\left( \begin{aligned} \cos\delta\\ \sin\delta\end{aligned} \right)$. (3)

Here, the externally applied electric field $\boldsymbol{E}$, is included in the integral $I_{J}(\boldsymbol{E})$. The factors $\gamma_{z}\gamma_{\perp}$ and $\Delta_{\perp}$ denote the phonon induced spin-dependent and spin-independent scattering processes, respectively.

A second condition that must be fulfilled is that the chiral bands are non-degenerate in the sense that there is an occupation imbalance between the bands. In the Hamiltonian, this is reflected by $\Delta_{z\boldsymbol{k}}$, which is included in the integral $I_{J}(\boldsymbol{E})$. Because of the occupation imbalance, the majority band contributes more to the current and since this band has a well-defined chirality, the electrons’ spins areoriented according to this chirality and thereby forced to bend into the transverse direction. The occupation imbalance between the bands can be interpreted as the electrons acquire a directional preference in their scattering off the phonons, as demonstrated in Fig. 5B.

The broken chiral degeneracy between the chiral bands leads to an induced effective magnetic moment. Under the same condition as above, the transverse components of this moment, pertaining to the anomalous Hall effect, can be expressed as

$\left( \begin{aligned} M_{x} \\ M_{y} \end{aligned} \right)=\gamma_{\perp}\Delta_{\perp}\Delta_{z}I_{M}(\boldsymbol{E})\left( \begin{aligned} \cos\delta\\ \sin\delta\end{aligned} \right)$. (4)

The presence of both $\gamma_{\perp}$ and $\Delta_{\perp}$ indicates that the same mechanisms, chirality and spin-orbit coupling, are responsible for the induced transverse moment as for inducing the spin polarization in the current. In addition, the broken degeneracy between the chiral bands is presented here explicitly through the parameter $\Delta_{z}$.

The previously introduced couplings are defined in the following fashion with respect to the electron-phonon coupling $\mathcal{H}_{\text{e-ph}}=\underset{\boldsymbol{kq}}{\sum}\Psi_{\boldsymbol{k}+\boldsymbol{q}}^{\dagger}H_{\boldsymbol{kq}}\Psi_{\boldsymbol{k}}(b_{\boldsymbol{q}}+b_{-\boldsymbol{q}}^{\dagger})$, where $b_{\boldsymbol{q}}+b_{-\boldsymbol{q}}^{\dagger}$ is the quantum nuclear displacement operator, whereas

$H_{\boldsymbol{kq}}=\left( \begin{matrix} U_{\boldsymbol{q}}\sigma^{0}+\boldsymbol{J}_{\boldsymbol{kq}}\cdot\boldsymbol{\sigma} & U_{12\boldsymbol{q}}\sigma^{0}+\boldsymbol{J}_{12\boldsymbol{kq}}\cdot\boldsymbol{\sigma} \\ U_{12\boldsymbol{q}}\sigma^{0}-\boldsymbol{J}_{12\boldsymbol{kq}}\cdot\boldsymbol{\sigma} & U_{\boldsymbol{q}}\sigma^{0}+\boldsymbol{J}_{\boldsymbol{kq}}\cdot\boldsymbol{\sigma} \end{matrix} \right)$, (5)

defines the intraband (diagonal) and interband (off-diagonal) electron-phonon couplings in terms of $2\times2$-matrices. The parameters $U_{\boldsymbol{q}}$, $U_{12\boldsymbol{q}}$, $\boldsymbol{J}_{\boldsymbol{kq}}$, and $\boldsymbol{J}_{12\boldsymbol{kq}}$ are defined in refs. ^[[1]](#endnote-1)^ and ^[[2]](#endnote-2)^. Specifically, the three component vectors $\boldsymbol{J}_{\boldsymbol{kq}}$, and $\boldsymbol{J}_{12\boldsymbol{kq}}$ account for phonon assisted spin-orbit coupling, due to a phonon assisted spin-exchange interaction. For more details about the definitions of these parameters, we refer to, e.g., Eq. (5) in ref. 1.

The spin exchange interaction, parametrized in $\boldsymbol{J}_{\boldsymbol{kq}}$, and $\boldsymbol{J}_{12\boldsymbol{kq}}$, is a result of the electronic polarizability of the system where the functions $\Pi_{0\boldsymbol{k}}$ and $\boldsymbol{\Pi}_{1\boldsymbol{k}}$ (defined below) account for polarizability due to electro-phonon interactions. To the first order in the phonon propagation, the chirality and interband spin-orbit couplings are defined by

$\Delta_{x\boldsymbol{k}}=2\underset{\boldsymbol{q}}{\sum}(U_{\boldsymbol{q}}U_{12\boldsymbol{q}}\Pi_{0\boldsymbol{kq}}+U_{12\boldsymbol{q}}\boldsymbol{J}_{\boldsymbol{kq}}\cdot\boldsymbol{\Pi}_{1\boldsymbol{kq}})$,

$\Delta_{y\boldsymbol{k}}=2\underset{\boldsymbol{q}}{\sum}(\boldsymbol{J}_{\boldsymbol{kq}}\cdot\boldsymbol{J}_{12\boldsymbol{kq}}\Pi_{0\boldsymbol{kq}}+U_{\boldsymbol{q}}\boldsymbol{J}_{12\boldsymbol{kq}}\cdot\boldsymbol{\Pi}_{1\boldsymbol{kq}})$, (6)

$\Delta_{z\boldsymbol{k}}=\underset{\boldsymbol{q}}{\sum}[(U_{\boldsymbol{q}}^{2}+J_{\boldsymbol{kq}}^{2}+U_{12\boldsymbol{q}}^{2}+J_{12\boldsymbol{kq}}^{2})\Pi_{0\boldsymbol{kq}}+2U_{\boldsymbol{q}}\boldsymbol{J}_{\boldsymbol{kq}}\cdot\boldsymbol{\Pi}_{1\boldsymbol{kq}}]$,

and

$\gamma_{x\boldsymbol{k}}=2\underset{\boldsymbol{q}}{\sum}U_{\boldsymbol{q}}U_{12\boldsymbol{q}}\boldsymbol{\Pi}_{1\boldsymbol{kq}}$,

$\gamma_{y\boldsymbol{k}}=2\underset{\boldsymbol{q}}{\sum}(U_{\boldsymbol{q}}\boldsymbol{J}_{12\boldsymbol{kq}}\Pi_{0\boldsymbol{kq}}-\boldsymbol{J}_{\boldsymbol{kq}}\cdot\boldsymbol{J}_{12\boldsymbol{kq}}\boldsymbol{\Pi}_{1\boldsymbol{kq}}+\boldsymbol{J}_{\boldsymbol{kq}}\cdot\boldsymbol{\Pi}_{1\boldsymbol{kq}}\boldsymbol{J}_{12\boldsymbol{kq}}+\boldsymbol{J}_{12\boldsymbol{kq}}\cdot\boldsymbol{\Pi}_{1\boldsymbol{kq}}\boldsymbol{J}_{\boldsymbol{kq}})$, (7)

$\gamma_{z\boldsymbol{k}}=2\underset{\boldsymbol{q}}{\sum}[U_{\boldsymbol{q}}\boldsymbol{J}_{\boldsymbol{kq}}\Pi_{0\boldsymbol{kq}}+\boldsymbol{J}_{\boldsymbol{kq}}\cdot\boldsymbol{\Pi}_{1\boldsymbol{kq}}\boldsymbol{J}_{\boldsymbol{kq}}-\boldsymbol{J}_{12\boldsymbol{kq}}\cdot\boldsymbol{\Pi}_{1\boldsymbol{kq}}\boldsymbol{J}_{12\boldsymbol{kq}}$

$+iU_{12\boldsymbol{kq}}\boldsymbol{J}_{12\boldsymbol{kq}}\times\boldsymbol{\Pi}_{1\boldsymbol{k}}+(U_{\boldsymbol{q}}^{2}-J_{\boldsymbol{kq}}^{1}+U_{12\boldsymbol{q}}^{2}-J_{12\boldsymbol{kq}}^{2})\boldsymbol{\Pi}_{1\boldsymbol{k}}]$,

respectively. These quantities are essentially energy dependent functions, given by the electron-phonon polarization loop:

$\Pi_{0\boldsymbol{kq}}(\omega)=\underset{s}{\sum}(\frac{n_{B}(\omega_{\boldsymbol{q}})+1-f(\varepsilon_{s\boldsymbol{k}})}{\omega-\varepsilon_{s\boldsymbol{k}}-\omega_{\boldsymbol{q}}+1/\tau_{\text{ph}}}+\frac{n_{B}(\omega_{\boldsymbol{q}})+f(\varepsilon_{s\boldsymbol{k}})}{\omega-\varepsilon_{s\boldsymbol{k}}+\omega_{\boldsymbol{q}}+1/\tau_{\text{ph}}})$,

(8)

$\boldsymbol{\Pi}_{1\boldsymbol{kq}}(\omega)=\overset{̂}{\boldsymbol{k}}\underset{s}{\sum}s(\frac{n_{B}(\omega_{\boldsymbol{q}})+1-f(\varepsilon_{s\boldsymbol{k}})}{\omega-\varepsilon_{s\boldsymbol{k}}-\omega_{\boldsymbol{q}}+1/\tau_{\text{ph}}}+\frac{n_{B}(\omega_{\boldsymbol{q}})+f(\varepsilon_{s\boldsymbol{k}})}{\omega-\varepsilon_{s\boldsymbol{k}}+\omega_{\boldsymbol{q}}+1/\tau_{\text{ph}}})$,

where $\varepsilon_{s\boldsymbol{k}}=\varepsilon_{\boldsymbol{k}}+s|\boldsymbol{\alpha}_{\boldsymbol{k}}|$, $s=\pm1$, whereas $\omega_{\boldsymbol{q}}$ denotes the phonon energy dispersion, and $n_{B}(\omega)$ and $f(\omega)$ are the Bose-Einstein and Fermi-Dirac distribution relations, respectively.

**Fig. S7:** Illustration of the polarization loop. Straight and wiggly lines denote electron and phonon propagators, respectively.

**References**

1. Fransson, J., Vibrational origin of exchange splitting and chiral-induced spin selectivity, 2020, *Phys. Rev. B*., **102**, 235416, [10.1103/PhysRevB.102.235416](https://dx.doi.org/10.1103/PhysRevB.102.235416) [↑](#endnote-ref-1)
2. Fransson. J., et al., Chiral phonon induced spin polarization, 2023, *Phys. Rev. Research*., **5**, L022039, [10.1103/PhysRevResearch.5.L022039](https://dx.doi.org/10.1103/PhysRevResearch.5.L022039) [↑](#endnote-ref-2)
